# Reconstruction of the Vancomycin-Susceptible *Staphylococcus aureus* Phenotype From a Vancomycin-Intermediate *S. aureus* XN108

**DOI:** 10.3389/fmicb.2018.02955

**Published:** 2018-11-28

**Authors:** Huagang Peng, Yifan Rao, Wenchang Yuan, Ying Zheng, Weilong Shang, Zhen Hu, Yi Yang, Li Tan, Kun Xiong, Shu Li, Junmin Zhu, Xiaomei Hu, Qiwen Hu, Xiancai Rao

**Affiliations:** ^1^Department of Microbiology, College of Basic Medical Sciences, Key Laboratory of Microbial Engineering Under the Educational Committee in Chongqing, Army Medical University (Third Military Medical University), Chongqing, China; ^2^Institute of Modern Biopharmaceuticals, School of Life Sciences, Southwest University, Chongqing, China; ^3^Department of Clinical Laboratory, The Fifth Affiliated Hospital of Guangzhou Medical University, Guangzhou, China

**Keywords:** Vancomycin-intermediate *Staphylococcus aureus*, vancomycin resistance, WalKR, GraSR, RpoB

## Abstract

The emergence of vancomycin-intermediate *Staphylococcus aureus* (VISA) has raised healthcare concerns worldwide. VISA is often associated with multiple genetic changes. However, the relative contributions of these changes to VISA phenotypes are incompletely defined. We have characterized VISA XN108 with vancomycin MIC of 12 μg/ml. Genome comparison revealed that WalK(S221P), GraS(T136I), and RpoB(H481N) mutations possibly contributed to the VISA phenotype of XN108. In this study, the above mutations were stepwise cured, and the phenotypes between XN108 and its derivates were compared. We constructed four isogenic mutant strains, XN108-WalK(P221S) (termed as K65), XN108-GraS(I136T) (termed as S65), XN108-RpoB(N481H) (termed as B65), and XN108-WalK(P221S)/GraS(I136T) (termed as KS65), using the allelic replacement experiments with the native alleles derived from a vancomycin-susceptible *S. aureus* isolate DP65. Antimicrobial susceptibility test revealed K65 and S65 exhibited decreased vancomycin resistance, whereas B65 revealed negligibly differed when compared with the wild-type XN108. Sequentially introducing WalK(P221S) and GraS(I136T) completely converted XN108 into a VSSA phenotype. Transmission electronic microscopy and autolysis determination demonstrated that cell wall thickening and decreasing autolysis were associated with the change of vancomycin resistance levels. Compared with XN108, K65 exhibited 577 differentially expressed genes (DEGs), whereas KS65 presented 555 DEGs. Of those DEGs, 390 were common in K65 and KS65, including those upregulated genes responsible for citrate cycle and bacterial autolysis, and the downregulated genes involved in peptidoglycan biosynthesis and teichoic acid modification. In conclusion, a VSSA phenotype could be completely reconstituted from a VISA strain XN108. WalK(S221P) and GraS(T136I) mutations may work synergistically in conferring vancomycin resistance in XN108.

## Introduction

*Staphylococcus aureus* is one of the most common pathogens causing hospital and community-associated infections worldwide (Boucher et al., 2010; Uhlemann et al., 2014; Kong et al., 2016). As one of the last-line antibiotics to treat *S. aureus* infections, vancomycin inhibits cell wall synthesis by binding to the D-alanyl–D-alanine carboxyl terminus of cell wall precursor molecules, thereby preventing the cross-linking of peptidoglycan (Walsh, 2000). However, its increased use in clinical practice has led to the emergence of VISA and hVISA strains, which are increasingly reported globally (Howden et al., 2010; Gardete and Tomasz, 2014; Hu et al., 2016; McGuinness et al., 2017).

VISA isolates usually exhibit several common features, such as thickened cell wall, reduced autolysis, impaired virulence, and vancomycin-intermediate resistance (Hu et al., 2016). However, the molecular mechanisms of VISA formation have not been fully elucidated. Diverse mutations that occur in genes encoding two-component systems (TCSs) play important roles in the formation of VISA, including VraSR (Mwangi et al., 2007), GraSR (Howden et al., 2008; Neoh et al., 2008), and WalKR (Howden et al., 2011; Shoji et al., 2011; McEvoy et al., 2013). WalKR regulates the genes involved in cell wall metabolism (Dubrac et al., 2008). The WalK(S221P), WalK(G223D) or WalR(K208R) mutations in vancomycin resistance have been verified experimentally using clinical or *in vitro* induced VISA isolates (Howden et al., 2011; Hu et al., 2015; Peng et al., 2017). Functionally, both WalK(S221P) and WalK(G223D) mutations exhibited a reduced autophosphorylation status of WalK, thereby decreasing the phosphorylation and transcriptional activity of WalR (Hu et al., 2015; Peng et al., 2017). GraSR confers vancomycin-intermediate resistance through upregulating *dltABCD* and *mprF* genes (Falord et al., 2011, 2012). GraR(N197S) is one of six mutations involved in VISA strain Mu50; GraS(T136I) mutation usually contributes to the formation of hVISA (Howden et al., 2008, 2011; Neoh et al., 2008). Other mutations in certain genes, including *msrR*, *sle1*, and *rpoB*, also play important roles in VISA formation. The RpoB(H481Y) mutation of the β subunit of bacterial RNA polymerase is often presented in VISA isolates to increase the vancomycin resistance level by 1–2 μg/ml (Cui et al., 2010; Matsuo et al., 2011; Watanabe et al., 2011).

A single mutation is usually not sufficient to fully convert a VSSA isolate to the VISA phenotype, and mutations in VISA may have cumulative effects in VISA formation (Hu et al., 2016). To determine the relative contribution of each mutation in VISA formation, the VISA phenotype of Mu50 was completely reconstituted by sequentially introducing mutations [VraS(S329L), MsrR(E146K), GraR(N197S), RpoB(H481Y), Fdh2(A297V), and Sle1(Δ67aa)] into six genes of the VSSA strain N315ΔIP (Katayama et al., 2016). However, genetic swapping is extensive work because several mutations usually exist in a VISA strain, and studies on the complete reconstitution or reversion of VISA are limited. We previously characterized an ST239 SCC*mec*-III VISA strain XN108 with 12 μg/ml vancomycin MIC (Zhang et al., 2013). Genome sequencing and comparisons showed that WalK(S221P), GraS(T136I), and RpoB(H481N) mutations might contribute to the VISA phenotype of XN108 (Peng et al., 2017). In this study, the above mutations were stepwise cured by introducing the WalK(P221S), GraS(I136T), and RpoB(N481H) alleles derived from a VSSA isolate DP65. The phenotypes, such as vancomycin susceptibilities, bacterial autolytic activities, and cell wall morphologies, between XN108 and its derivates were compared. The differentially expressed genes (DEGs) among XN108 and its derivates were analyzed after RNA-seq.

## Materials and Methods

### Bacterial Strains, Plasmids, and Growth Conditions

The *S. aureus* strains and plasmids used in this study are listed in Supplementary Table S1. The cloning and transformation of *Escherichia coli* DH5α were carried out by standard techniques (TianGen Biotech Co., Ltd., Beijing, China). All *S. aureus* strains were cultivated in brain heart infusion (BHI) broth or tryptic soy broth (TSB, Oxford) with aeration at 37°C, unless indicated otherwise. The antibiotic chloramphenicol (Sigma Chemical Co., St. Louis, MO, United States) 20 mg/ml was used for selection of the *S. aureus* transformants.

### Antibiotic Susceptibility Tests

Antibiotic susceptibility was determined by E-test with a vancomycin strip according to instructions provided by the manufacturer (bioMérieux, France). The population analysis profile (PAP) method was conducted as previously described (Wootton et al., 2001). Briefly, the vancomycin PAP was determined by serial dilution of an overnight BHI culture and by inoculation of BHI agar (BHIA) containing 0–12 μg/ml of vancomycin. Colonies were counted after incubation for 48 h at 37°C and plotted as numbers of CFU/ml vs. the vancomycin concentration.

### Construction of K65, S65, B65, and KS65 Mutants

All allelic replacement strains tested in this study are described in Supplementary Table S1. The candidate genes WalK(P221S), GraS(I136T), and RpoB(N481H) from a VSSA isolate DP65 which has identical molecular types (ST239 and SCC*mec*-III) to XN108 (Supplementary Table S1) were amplified using primers listed in Supplementary Table S2 and sequentially introduced into XN108 with an allelic replacement strategy as described (Peng et al., 2017). The resulting strains XN108-WalK(P221S), XN108-GraS(I136T), XN108-RpoB(N481H), and XN108-WalK(P221S)/GraS(I136T) were designated as K65, S65, B65, and KS65, respectively. The allelic replacement of each gene was confirmed by DNA sequencing.

### Growth Curve

The wild-type VISA strain XN108 and its isogenic derivates were incubated overnight in 2 ml TSB at 37°C with shaking at 200 rpm. The overnight cultures were diluted in 100 ml fresh TSB to obtain the same starting optical density (OD) at 600 nm. The growth of each strain was added to the wells of a 96-well microtiter plate monitored by microplate reader (SpectraMax^®^M2/M2e, United States) at 1 h intervals for a total of 12 h.

### Triton X-100 Stimulated Autolysis

Triton X-100 stimulated autolysis was performed as previously described with some modifications (Xue et al., 2013). Briefly, the strains were grown in TSB to early exponential phase (OD600 = 1.0) at 37°C with shaking. Bacterial cells were collected by centrifugation, washed twice with 0.05 M Tris–HCl buffer (pH 7.5), resuspended in an equal volume of Tris*–*HCl buffer containing 0.05% (v/v) Triton X-100, and incubated at 37°C with shaking. The decrease in the optical density at 600 nm (OD600) was measured every hour using a microplate reader. The experiment was repeated at least three times.

### Transmission Electron Microscopy (TEM)

*S. aureus* strains were cultured in TSB for 24 h at 37°C with shaking. Samples were prepared as previously described (Yuan et al., 2013) and observed under a TECNAI10 transmission electron microscope (Philips, Netherlands). Cell wall thickness was determined in four separate quadrants of each cell, and 60 cells with nearly equatorial cut surfaces were chosen from each strain for determination. The results are expressed as means ± standard deviations (SD) and the data were evaluated using Student’s *t*-test, where a *P*-value < 0.05 was considered statistically significant. All statistical tests were two-tailed.

### RNA-seq Analysis and RT-qPCR

For RNA-seq analysis, the total RNA was isolated from XN108, K65, and KS65 as previously described (Yuan et al., 2013), and three biological replicates were prepared for RNA sequencing. Library construction and Illumina sequencing was performed at Novogene Bioinformatics Technology Co., Ltd. (Beijing, China). RNA-seq analysis was performed according to the protocol recommended by the manufacturer (Illumina Inc.). Differentially expressed gene analysis was performed using the DESeq R package (1.10.1). For RT-qPCR detection, the first cDNA was synthesized using a RevertAid First Strand cDNA Synthesis Kit (Thermo Fisher Scientific, United States) from 500 ng of total RNA with random primers in a 20 μl reaction mixture. qPCR was performed with SsoAdvanced^TM^ Universal SYBR^®^Green Supermix kit (Bio-Rad, United States) using the Bio-Rad CFX96^TM^ Realtime detection system (Bio-Rad, United States). The primers used are listed in Supplementary Table S2. The 16S rRNA gene was selected as the reference gene for normalization. The assays were repeated with three independent biological samples. Statistical analyses were performed based on the Student’s *t*-test to determine the significance of the gene expression levels, where *P* < 0.05 was considered statistically significant.

### Construction of LacZ Reporter

To construct the reporter plasmid pOS-*dlt* for detection of the effect of GraS(T136I) on *dlt* operon expression, a 442-bp fragment of the *dlt* operon promoter regions was amplified from *S. aureus* XN108 genomic DNA with the primers pOS*dlt*-lacZ-5 and pOS*dlt*-lacZ-3 (Supplementary Table S2). The fragment was digested with *Bam*HI/*Eco*RI and then cloned into the shuttle vector pOS1 to generate reporter plasmid pOS-dlt. After transformed into *S. aureus* RN4220, the pOS-dlt was subsequently transformed into *S. aureus* K65 and KS65, respectively.

### β-Galactosidase Activity Assay

The stationary-phase cultures of the K65 and KS65 carrying LacZ reporter plasmid pOS-dlt were diluted 1:100 into TSB with chloramphenical (Sigma Chemical Co., St. Louis, MO, United States), 20 mg/ml. Cells were collected at the early log phase (OD600 of 1.0) and lysed for 30 min at 37°C in 100 μl ABT LSA buffer (60 mM K_2_HPO_4_, 40 mM KH_2_PO_4_, 100 mM NaCl, 0.01% Triton X-100, 50 μg/ml lysostaphin). We then added 100 μl of ABT buffer and 100 μl of 4 μg/ml ONPG (onitrophenyl-β-D-galactopyranoside) to initiate the reaction. The samples were incubated at 37°C until a yellow color became apparent, and then 1 ml Na_2_CO_3_ was added to stop the reaction. The enzyme activities were expressed as Miller units. All samples were tested in triplicate.

## Results

**FIGURE 1:**
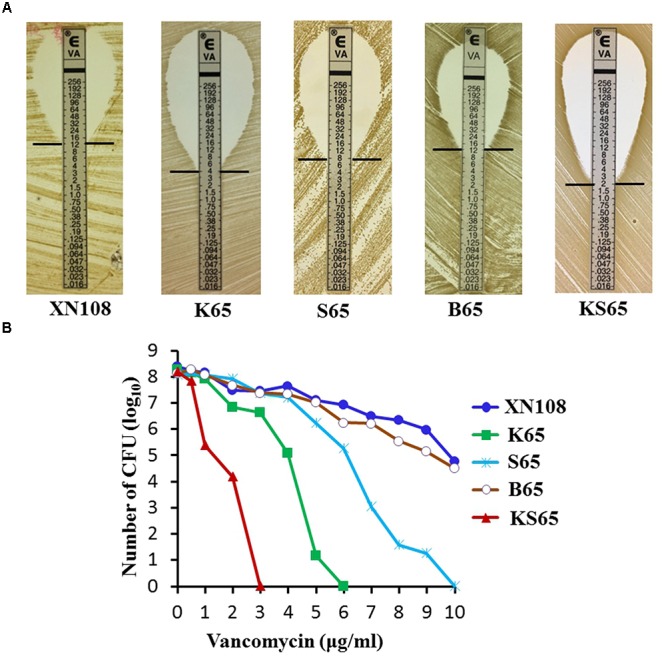
Vancomycin susceptibilities of VISA XN108 and its derivates. **(A)** E-test of vancomycin susceptibilities of XN108, K65, S65, B65, and KS65. **(B)** Population analysis of the vancomycin susceptibility profiles of XN108, K65, S65, B65, and KS65.

Sequentially Introducing WalK(P221S) and GraS(I136T) Sufficiently Convert XN108 to the VSSA Phenotype XN108 is an ST239 and SCC*mec*-III VISA isolate (vancomycin MIC = 12 μg/ml) characterized in our previous study (Zhang et al., 2013). Genome sequencing and comparison revealed that WalK(S221P), GraS(T136I), and RpoB(H481N) might contribute to the VISA phenotype of XN108 (Peng et al., 2017). Substitution of *walK* in the VSSA strain N315 with that from XN108 increased the vancomycin MIC of N315 from 1.5 μg/ml to 8 μg/ml, whereas replacement of *walK* in the XN108 with that from N315 decreased the vancomycin MIC of XN108 from 12 μg/ml to 4 μg/ml (Peng et al., 2017). However, *walK* from N315 has other mutations including WalK(R222K) and WalK(A468T), in addition to WalK(P221S), as compared with that of XN108 (Supplementary Figure S1). To avoid the effect of extra mutations on drug resistance, the *walk*, *graS*, and *rpoB* genes from the VSSA isolate DP65, which has identical molecular types (ST239 and SCC*mec*-III) to XN108 and no additional site mutations, were used as WalK(P221S), GraS(I136T), and RpoB(N481H) candidate genes and independently introduced into XN108 through an allelic replacement strategy. The allelic replacement of each gene was confirmed by DNA sequencing (Supplementary Figure S2). The resulting strains XN108-WalK(P221S), XN108-GraS(I136T), XN108-RpoB(N481H), and XN108-WalK(P221S)/GraS(I136T) were designated as K65, S65, B65, and KS65, respectively. Growth curve determination revealed that the growth rate of K65, S65, or KS65 increased slightly compared with that of XN108 (Supplementary Figure S3). Antimicrobial susceptibility test demonstrated that K65 had a vancomycin MIC of 4 μg/ml (Figure 1A), which was equal to that of XN108-WalK(P221S-R222K-A468T) constructed using *S. aureus* N315 *walK* (Peng et al., 2017), suggesting that the WalK(R222K) and WalK(A468T) mutations in N315 may not affect the vancomycin resistance. S65 had a vancomycin MIC of 8 μg/ml, while B65 had a vancomycin MIC of 12 μg/ml (Figure 1A), suggesting that the GraS(T136I) mutation also contributes to vancomycin resistance in XN108 whereas the RpoB(H481N) mutation has limited role. Both K65 and S65 presented a VISA phenotype in accordance with the standards of the CLSI (2016). Continuously restoring GraS(T136I) in K65 with GraS(I136T) from DP65 resulted in KS65 to be a VSSA strain with vancomycin MIC of 2 μg/ml (Figure 1A). Population analysis also showed that independent or sequential introduction of WalK(P221S), GraS(I136T) into VISA XN108 decreased vancomycin resistance gradually (Figure 1B).

### K65 and KS65 Exhibited Decreased Cell Wall Thickening and Increased Autolysis Compared With XN108

**FIGURE 2:**
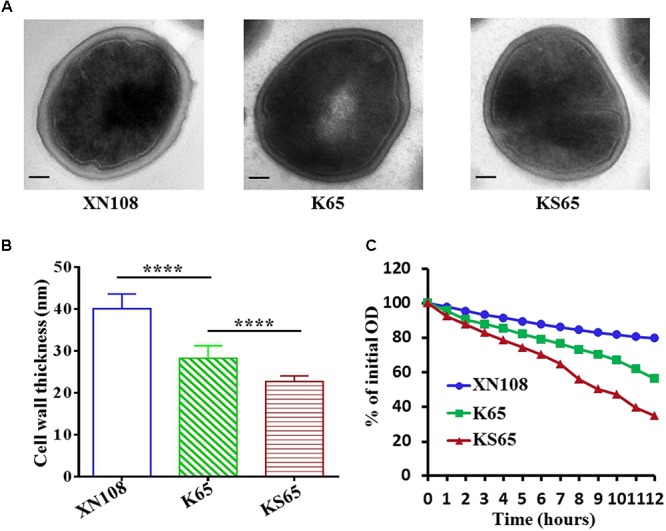
Transmission electron microscopy and autolytic analysis of the XN108 and its derivates. **(A)** Morphological features of VISA XN108 and its derivates K65 and KS65 observed under a transmission electron microscope (magnification, × 120,000; bar = 100 nm). **(B)** Comparison of cell wall thickness of XN108, K65 and KS65. Data are expressed as the mean ± SD for 60 cells of each strain. Differences were evaluated by Student’s *t*-test, and all statistical tests were two-tailed. ^∗∗∗∗^*P* < 0.0001. **(C)** Triton X-100 stimulated autolytic analysis. Bacterial cells were grown in BHI broth to mid-logarithmic phase, pelleted, washed twice with ice-cold water, and then analyzed as described in Methods. The percentage of the remaining optical density of each strain at each time point was plotted. The test was repeated 3 times, and 1 representative was indicated.

A VISA strain usually has a thickened cell wall, which is considered to be associated with the clogging mechanism of vancomycin resistance (Cui et al., 2003; Hu et al., 2016). VISA XN108 exhibited a thickened cell wall (Figure 2A; Zhang et al., 2013). We determined whether the reduced vancomycin resistance in the derivates of XN108 is associated with the decrease in cell wall thickness. Transmission electron microscopy revealed that K65 presented significantly decreased cell wall thickness (28.3 ± 2.9 nm) compared with XN108 (40.1 ± 3.5 nm; *P* < 0.0001). KS65 showed a normal *S. aureus* cell wall thickness (22.7 ± 1.4 nm), which is much thinner than that of K65 (Figures 2A,B). In addition, VISA strains often exhibit reduced autolysis (Hu et al., 2016). Autolytic analysis stimulated by Triton X-100 demonstrated that the autolytic activities of K65 and KS65 were gradually increased compared with that of XN108 (Figure 2C).

### Comparison of Gene Expression Profiles Among XN108, K65, and KS65

**FIGURE 3:**
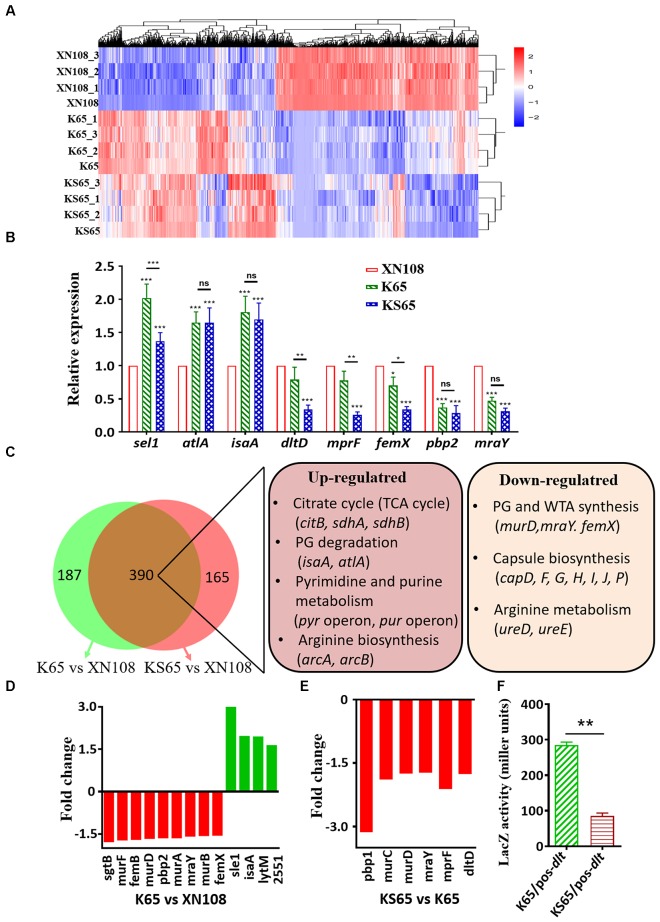
Transcriptional profiles of XN108, K65, and KS65. **(A)** Pairwise Euclidean distance with respect to the transcriptional profiles from each biological repeated sample. **(B)** RT-qPCR detection of the expression levels of indicated genes in XN108, K65, and KS65. Data from one experiment with three biological replicates were presented as mean ± SD. Expression of each gene of interest in XN108 was normalized to the 16S RNA gene expression and adjusted to 1.0, and their relative expressions in K65 and KS65 strains were indicated. ^∗^*P* < 0.05, ^∗∗^*P* < 0.01, and ^∗∗∗^*P* < 0.001, and ns represents no significance. **(C)** Schematic representation of the expression altered genes in K65 vs. XN108 (green) and KS65 vs. XN108 (pink). A total of 390 genes were commonly changed, and the major upregulated and downregulated genes are involved in certain biological pathways in KEGG analysis. **(D)**The fold change of the upregulated (red) and downregulated (green) genes responsible for cell wall biosynthesis and bacterial autolysis in K65 vs. XN108. **(E)** The fold change of the downregulated genes responsible for cell wall biosynthesis and modification in KS65 vs. XN108. **(F)** β-galactosidase assay. The pOS-*dlt* reporter plasmid was transformed into K65 and KS65, respectively. The LacZ activity was detected and represented as mean ± SD (*n* = 3). ^∗∗^*P* < 0.01.

Although numerous mutations in certain genes are associated with VISA phenotypes, their mechanisms on promoting cell wall biosynthesis and impairing bacterial autolysis are incompletely defined (Howden et al., 2010; Hu et al., 2016). The above results showed that the introduction of WalK(P221S) and GraS(I136T) into VISA XN108 completely restored the VSSA phenotype and prompted us to study how WalK(S221P) and GraS(T136I) mutations alter gene expression profiles in VISA strains. Thus, RNA-seq analysis was performed on XN108, K65, and KS65. The differentially expressed genes (DEGs) in the three independent biological repeats were illustrated by Euclidean distance calculation and showed global alteration in gene expression profiles between XN108 and its derivates (Figure 3A). Eight significantly upregulated (*sel1*, *atlA*, and *isaA*) and downregulated (*dltA*, *mprF*, *femX*, *pbp2*, and *mraY*) genes in the XN108 derivates, compared with those in XN108, were selected. Their expression levels in K65, KS65, and XN108 were verified by RT-qPCR. The RT-qPCR results were consistent with those obtained after RNA-seq (Figure 3B).

A gene expression determined by RNA-seq and demonstrated a fold change ≥ 1.5 with an adjusted *P* < 0.05 was included in the analysis. Compared with XN108, K65 with reduced vancomycin resistance had 299 upregulated and 278 downregulated genes (Supplementary Table S3), whereas KS65 with a vancomycin-susceptible phenotype had 260 upregulated and 295 downregulated genes (Supplementary Table S4). However, KS65 had only 42 upregulated and 73 downregulated genes compared with K65 (Supplementary Table S5). WalK(S221P) mutation that plays crucial roles in conferring VISA phenotype in XN108 (Peng et al., 2017) altered more gene expressions than GraS(T136I) did. In total, 390 changed genes in K65 vs. XN108 (67.7%, 390/577) were identical to those in KS65 vs. XN108 (70.3%, 390/555), including those common upregulated genes responsible for citrate cycle, bacterial autolysis, pyrimidine and purine metabolisms, and arginine biosynthesis, and the downregulated genes involved in peptidoglycan, teichoic acid, and capsule biosynthesis, and arginine metabolism based on KEGG analysis (Figure 3C).

WalKR is a master regulon for cell wall metabolism in *S. aureus* (Dubrac et al., 2008). WalR regulates *S. aureus* by directly binding to the promoter regions of certain autolysis-associated genes, such as *atlA*, *lytM*, *isaA*, and *ssaA* (Dubrac and Msadek, 2004). RNA-seq revealed that the expression levels of *atlA*, *isaA*, *SAXN108_2551*, and *lytM* were upregulated in K65 compared with those in XN108 (Figure 3D and Supplementary Table S3). GraR binds to the promoter regions of *dltABCD* and *mprF* to initiate their transcription (Falord et al., 2011). RNA-seq also revealed that the expression levels of *dltD* and *mprF* were downregulated in KS65 compared to K65 (Figure 3E). When transformed with a LacZ fusion reporter plasmid pOS-dlt, the β-galactosidase activities in KS65 were significantly decreased compared with those in K65 (*P* < 0.01, Figure 3F). This result suggested that GraS(T136I) mutation promoted the promoter activity of *dlt* operon, whereas WalK(S221P) mutation decreased WalKR activity to regulate its downstream genes (Hu et al., 2016; Peng et al., 2017).

## Discussion

With the increasing use of vancomycin, VISA infections pose a global threat (Walsh, 2000; Hu et al., 2016). VISA is often associated with multiple genetic changes, but a limited number of mutations in several genes contributing to VISA formation were evaluated, and the regulatory mechanism of vancomycin resistance in VISA strains has not been fully explored (Hu et al., 2016). Several mutations in VISA may work synergistically to promote its common phenotypes, and understanding the relative contribution of each mutation to VISA formation would help in determining the key molecular pathway linking the multiple genetic variations and the common downstream characteristics of VISA. In this study, we performed allelic exchange experiments to independently or stepwise revert the WalK(S221P), GraS(T136I), and RpoB(H481N) mutations in VISA XN108 and assessed the contributions of these mutations to vancomycin susceptibility and other VISA phenotypes. Our data revealed that WalK(S221P) and GraS(T136I) mutations played crucial roles in conferring the VISA phenotype of XN108, whereas RpoB(H481N) had negligible role in the VISA formation of XN108 (Figures 1, 2). RpoB(H481Y) mutation is one of the regulatory mutations increasing the level of resistance to vancomycin, daptomycin, and β-lactams (Katayama et al., 2016). The RpoB(H481Y) mutation exerts a stringent response in *S. aureus* whereas the role of RpoB(H481N) is not known (Gao et al., 2013). RpoB(H481N) mutation is usually associated with the fitness cost compensation mutation of RpoB(L466S) in the ST239-t030 linage (Shang et al., 2016). In XN108, RpoB(H481N) mutation is indeed associated with RpoB(L466S) mutation. Overall, we showed that RpoB(H481N) in XN108 was unlike RpoB(H481Y), which plays an important role in the VISA formation of Mu50. Thus, the exact function of RpoB(H481N) needs further investigation.

At the genetic level, the reversion of WalK(S221P) together with GraS(T136I) sufficiently convert XN108 to the VSSA phenotype. Mutations in both WalKR and GraSR TCSs are major evolutionary pathways to generate VISA, such as WalK(G223D) and GraS(T136I) in VISA JKD6008 compared with its VSSA parent strain JKD6009 (Howden et al., 2011). However, the mechanism of mutations in WalKR and GraSR that promote the common VISA phenotypes remains unclear. Our RNA-seq analysis revealed that the WalK(S221P) reverted strain K65 and the WalK(S221P)/GraS(T136I) reverted strain KS65 exhibited 390 common DEGs compared with XN108, including those upregulated genes responsible for citrate cycle, bacterial autolysis, pyrimidine and purine metabolisms, and arginine biosynthesis, and the downregulated genes involved in peptidoglycan synthesis, teichoic acid modification, capsule biosynthesis, and arginine metabolism (Figure 3C). Specific and reversible metabolic alterations are usually observed in genetically distinct VISA strains (Nuxoll et al., 2012; Alexander et al., 2014), suggesting that multiple genetic changes in VISA may alter certain metabolic pathways to contribute to common VISA phenotypes.

WalKR is the only essential TCS for viability in *S. aureus*, and it acts as an information conduit between extracellular structures and intracellular processes required for cell wall metabolism (Dubrac et al., 2008; Peng et al., 2017). Mutations in WalKR observed in VISA strains usually result in reduced WalKR function (Hu et al., 2015; Peng et al., 2017). RT-qPCR and RNA-seq analyses revealed that the downstream genes of WalKR, such as *sel1* and *atlA*, which are responsible for autolysis, were significantly upregulated, whereas those for cell wall biosynthesis, such as *femX* and *pbp2*, were significantly downregulated in WalK(S221P) reverted K65 and KS65 compared with those in XN108 (Figures 3B,C). GraSR is a TCS that is involved in glycopeptide resistance by modulating the D-alanylation of teichoic acids and lysylination of phosphatidylglycerol to increase the surface positive charges (Falord et al., 2012). Mutations in the GraSR of VISA strains often increase GraSR function (Hu et al., 2016). LacZ reporter assay showed that the *dlt* promotor controlled β-galactosidase activities in GraS(T136I) cured strain KS65 were significantly decreased compared with those in K65 (Figure 3E). Simultaneously, the downstream genes of GraSR, such as *pbp1*, *mprF*, and *dltD*, were significantly downregulated in the GraS(T136I) reverted KS65 compared with those in XN108 (Figures 3B–D). The RT-qPCR data revealed that GraSR regulon could overlap with the WalKR regulon in mediating cell wall synthesis. The reversion of WalK(S221P) resulted in the significantly down-regulation of *femX*, and sequentially cured GraS(T136) further decreased *femX* expression in KS65 (Figure 3B). This phenomenon was also observed by other investigators (Falord et al., 2011). Taken together, mutations in both WalKR and GraSR could promote decreased autolysis and thickened cell wall, thereby mediating vancomycin resistance in VISA.

**FIGURE 4:**
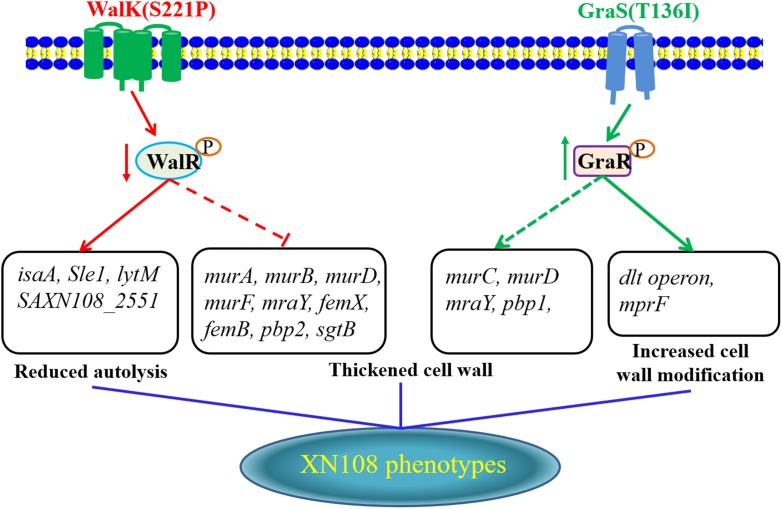
Proposed model for how the WalK(S221P) and GraS(T136I) mutations regulate vancomycin resistance in XN108.

In conclusion, adopting the allelic replacement strategy, a VSSA phenotype of the VISA XN108 was completely reconstructed by sequential introduction of WalK(P221S) and GraS(I136T) to cure certain mutations. Here, we proposed a model for the interpretation of vancomycin resistance in VISA XN108 (Figure 4). On the one hand, the mutated and reduced activity of the WalKR system decreased the expression of autolysins and increased *pbp2*, thereby decreasing autolysis and increasing the cell wall synthesis of XN108. On the other hand, the mutated and activated GraSR system increased the transcription of genes for cell wall synthesis and modification, including *pbp1*, *dltABCD*, and *mprF*, thereby increasing the cell wall biosynthesis and modification. Both WalK(S221P) and GraS(T136I) work synergistically to activate cell wall synthesis, promote cell wall modification, and decrease bacterial autolysis, which are all common features of VISA.

## Author Contributions

XR and QH conceived the study. HP, YR, WY, and YZ performed the experiments. HP, WS, YY, and QH analyzed the data. ZH, LT, KX, SL, and JZ contributed to reagents and materials. HP, XH, QH, and XR wrote the paper.

## Conflict of Interest Statement

The authors declare that the research was conducted in the absence of any commercial or financial relationships that could be construed as a potential conflict of interest.

## Abbreviations

B65: XN108-RpoB(N481H)
CLSI: clinical and laboratory standards institute
DEG: differential expressed gene
hVISA: heterogeneous VISA
K65: XN108-WalK(P221S)
KS65: XN108-WalK(P221S)/GraS(I136T)
MIC: minimum inhibitory concentration
S65: XN108-GraS(I136T)
VISA: vancomycin-intermediate *S. aureus*
VSSA: vancomycin-susceptible *S. aureus*
