# Supplementary material for: Reconstruction of the Vancomycin-Susceptible Staphylococcus aureus Phenotype From a Vancomycin-Intermediate S. aureus XN108
Source: Front Microbiol. 2018 Nov 28;9:2955. doi: 10.3389/fmicb.2018.02955 (PMC6279853; doi:10.3389/fmicb.2018.02955)
Supplement: Supplementary file 1 [file Data_Sheet_1.PDF]

Supplementary Material

Reconstruction of the vancomycin-susceptible *Staphylococcus aureus* phenotype from a vancomycin-intermediate *S. aureus* XN108

Huagang Peng *

*** Correspondence:** Xiancai Rao: raoxiancai@126.com
Qiwen Hu: huqiwen2004@163.com

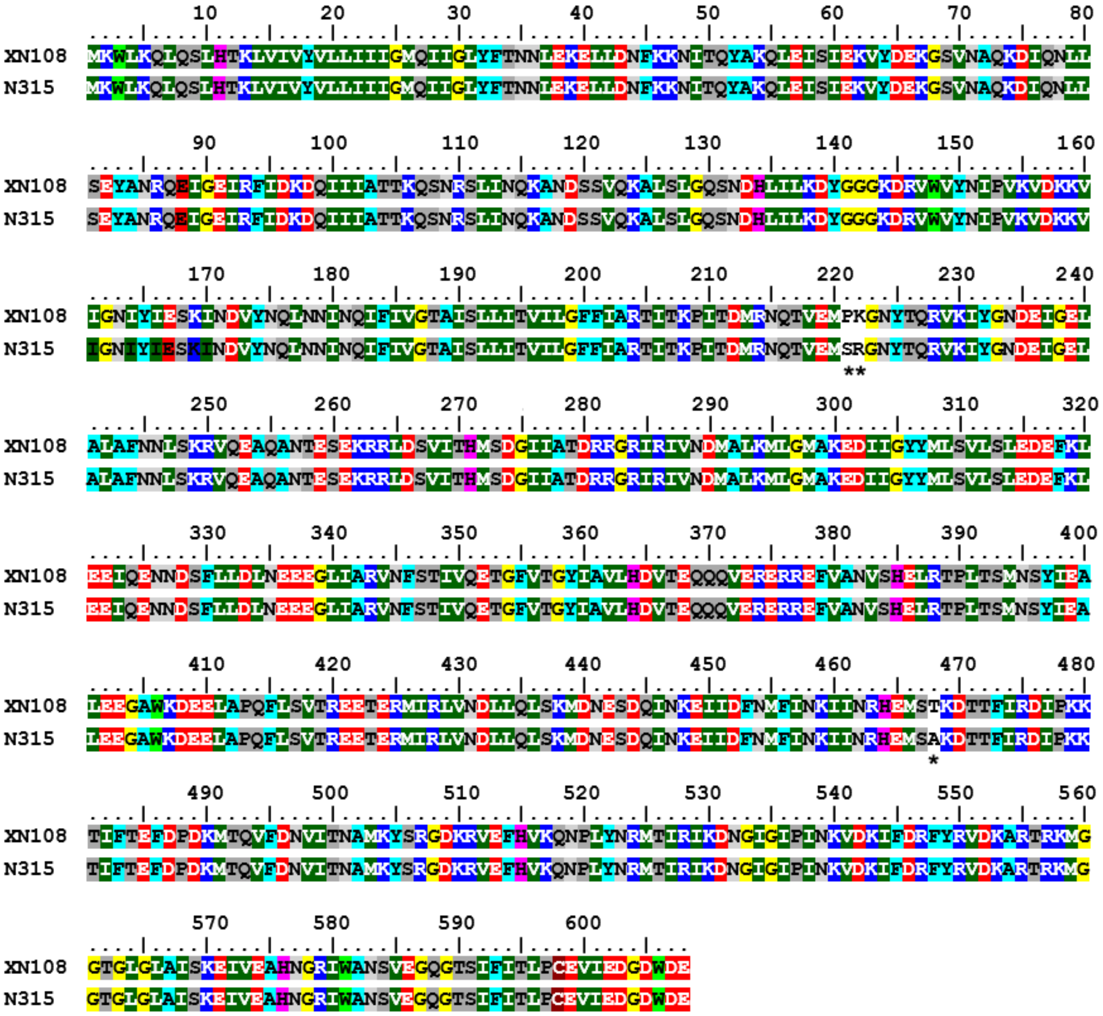

Figure S1. Alignment of WalK in XN108 and N315. The mutations in N315 versus XN108 were indicated by black stars.

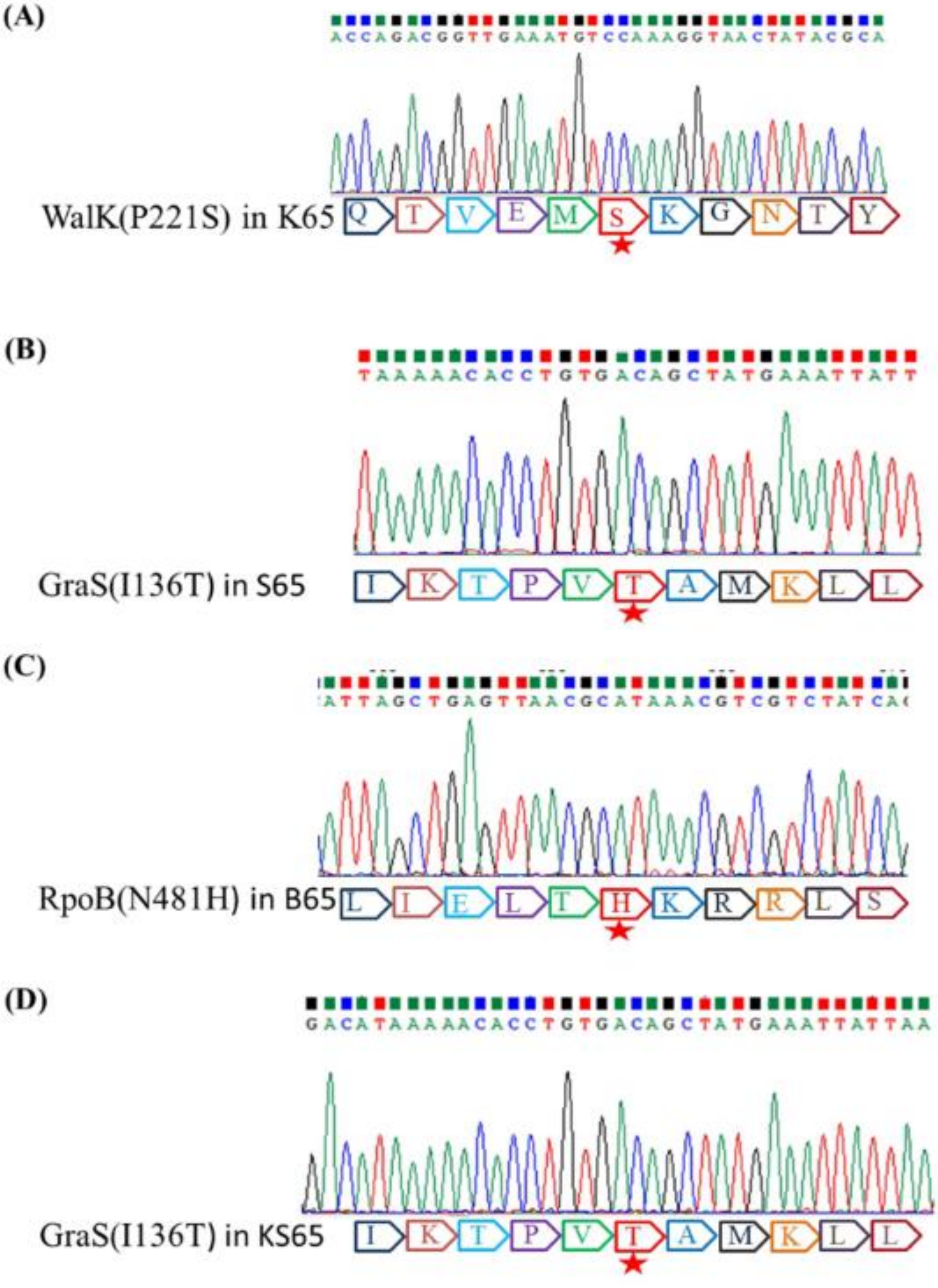

Figure S2. The reverted genes of *walK* (A), *graS* (B), and *rpoB* (C) and *graS* (D) in the K65, S65, B65, and KS65 strains, respectively. The cured sites were indicated by red stars.

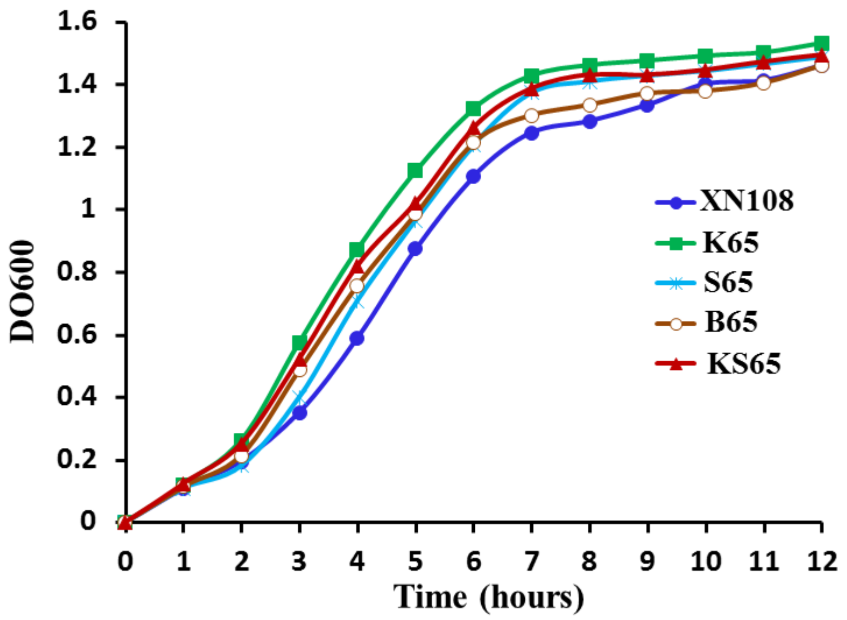

Figure S3. Growth curve determination. The overnight cultures were diluted in 100 ml fresh TSB to obtain the same starting optical density (OD) at 600 nm. The growth of each strain was determined by adding the culture to the wells in a 96-well microtiter plate monitored by microplate reader (SpectraMax®M2/M2e, USA) at 1 h intervals for a total of 12 h.

Table S1. Bacterial strains used in this study

Strain/Plasmid	Comments	Reference
Staphylococcus aureus		
DP65	VSSA clinical strain with ST239-SccmeC-III	This study
RN4220	derivative of strain NCTC8325-4, phage-cured, R-M-S negative	Berscheid A et al. (2012)
XN108	VISA clinical strain with GraS(T136I), WalK(S221P), and RpoB(H481N) mutations	Zhang X et al. (2013)
K65	allelic replacement WalK(P221S) in XN108	This study
KS65	allelic replacement GraS(I136T) in K65	This study
KSB65	allelic replacement RpoB(N481H) in KS65	This study
Escherichia coli		
DH5α	Clone host strain	
Plasmids		
pBT2	*E. coli* - *S. aureus* shuttle vector, vector for allelic exchange	Bruckner R et al (1997)
pBT2- walK(P221S)	pBT2 with *walk* loci from DP65	This study
pBT2- GraS(I136T)	pBT2 with *graS* loci from DP65	This study
pET2-RpoB(N481H)	pBT2 with *rpoB* loci from DP65	This study

References:

Berscheid A, Sass P, Weber-Lassalle K, Cheung AL, Bierbaum G. Revisiting the genomes of the *Staphylococcus aureus* strains NCTC 8325 and RN4220. *Int. J. Med. Microbiol.* 2012; 302: 84-7.

Zhang X, Hu Q, Yuan W, Shang W, Cheng H, Yuan J, et al. First report of a sequence type 239 vancomycin-intermediate *Staphylococcus aureus* isolate in Mainland China. *Diagn Microbiol. Infect. Dis.* 2013; 77: 64-8.

Bruckner R. Gene replacement in *Staphylococcus carnosus* and *Staphylococcus xylosus*. FEMS Microbiol. Lett. 1997; 151: 1-8.

Table S2. Primers used in this study

Primers	Sequence(5'-3')	Notes
pBT2-walK-5'	gcaggtcgactctagatgcaaatggctagaaaagt	WalK allelic replacement
pBT2-walK-3'	cggtacccggggatctaaaatgacagatttaatat	WalK allelic replacement
pBT2-graS-5'	ccggaattcactaaatgatattgggtgatatgg	GraS allelic replacement
pBT2-graS-3'	gcgggatccgtatatcagataattccttgtttg	GraS allelic replacement
pBT2-rpoB-5'	cggggtaccagaacgtgtaatcgtatctca	RpoB allelic replacement
pBT2-rpoB-3'	cgggtcgacatgtaacaatctttcttcgg	RpoB allelic replacement
isaA-RT-5’	ttgagaaaccagcagtttgacc	qRT-PCR
isaA-RT-3’	catcattagcagtggcattaggt	qRT-PCR
sle1-RT-5’	ctacgaactcaggatctgcaaca	qRT-PCR
sle1-RT-3 ’	cccagtttattagcattccacca	qRT-PCR
atlA-RT-5’	tgttgatggcttaggtattggtg	qRT-PCR
atlA-RT-3’	ttgggttaaagaaggcgatgt	qRT-PCR
femX-RT-5’	cgtggttggatgtagtcttttga	qRT-PCR
femX-RT-3’	acggtgaatggcagggtgttg	qRT-PCR
pbp2-RT-5’	ggtgggaaatccaactcaaaa	qRT-PCR
pbp2-RT-3’	cagaaccaaatccaccagtcaa	qRT-PCR
mraY-RT-5’	attcgagaagaaggtccacaaag	qRT-PCR
mraY-RT-3’	cgtcacaaataataacagtatgattgg	qRT-PCR
dltD-RT-5’	acgcagttgaacttgcatcacag	qRT-PCR
dltD-RT-3’	ctactggaacatttgattaatttgag	qRT-PCR
mprF-RT-5’	taatcacagtggcgacattcttc	qRT-PCR
mprF-RT-3’	ctcattcttactgggcgtttca	qRT-PCR
pos*dlt*-lacZ-5’	ccggaattcagtatggtttttaatcattggt	Reporter
pos*dlt*-lacZ-3’	cgcggatccggtttacttttagatttcat	Reporter
